# Three Years On: The Role of Pegcetacoplan in Paroxysmal Nocturnal Hemoglobinuria (PNH) since Its Initial Approval

**DOI:** 10.3390/ijms25168698

**Published:** 2024-08-09

**Authors:** Regina Horneff, Barbara Czech, Michael Yeh, Elena Surova

**Affiliations:** 1 Swedish Orphan Biovitrum AB, 171 65 Stockholm, Sweden; 2 Apellis Pharmaceuticals, Inc., Waltham, MA 02451, USA

**Keywords:** paroxysmal nocturnal hemoglobinuria, PNH, pegcetacoplan, complement, C3, proximal inhibitor

## Abstract

Paroxysmal nocturnal hemoglobinuria (PNH) is a rare disease characterized by complement-mediated hemolysis and potentially life-threatening complications. Pegcetacoplan, an inhibitor of complement components C3 and C3b, was approved by the US Food and Drug Administration (FDA) and European Medicines Agency (EMA) in 2021. A recent expansion to its indication by the EMA has made pegcetacoplan available for the treatment of both complement inhibitor-naïve and -experienced patients with PNH who have hemolytic anemia, a similarly broad patient population as in the US. This approval was based on results from the Phase 3 PEGASUS study, where pegcetacoplan showed superiority over the C5 inhibitor eculizumab with regard to improving the hemoglobin level in patients with anemia despite eculizumab treatment, and the Phase 3 PRINCE study, where pegcetacoplan showed superiority over supportive care with regard to hemoglobin stabilization and improving the lactate dehydrogenase level in complement inhibitor-naïve patients. In light of this recent indication expansion by the EMA, this article describes how the strong efficacy of pegcetacoplan is linked to its mechanism of action, which provides broad hemolysis control over both intravascular and extravascular hemolysis to improve a range of disease markers and enhance patients’ quality of life. Furthermore, additional data and learnings obtained from over 3 years of experience with pegcetacoplan are summarized, including long-term efficacy and safety results, real-world clinical experiences, pharmacokinetic characteristics, and extensive practical guidance for the first-to-market proximal complement inhibitor for PNH.

## 1. The Burden of PNH Remains High in Untreated Patients and in Many Patients Treated with C5 Inhibitors: Introduction

Paroxysmal nocturnal hemoglobinuria (PNH) is a rare hematological disease characterized by sudden episodes or ‘paroxysms’ of hemolysis, which can affect a diverse patient population [1,2]. PNH is caused when hematopoietic stem cells (HSCs) acquire a mutation in *PIGA*, giving rise to a persistent pool of mature blood cells harboring the same mutation. PIGA is required for the synthesis of glycosylphosphatidylinositol (GPI) anchors, necessary for the attachment of CD55 (also known as Complement Decay-Accelerating Factor) and CD59 (also known as Membrane Attack Complex [MAC]-Inhibitory Protein), two key GPI-anchored regulators of the complement system, to blood cell surfaces [1].

The loss of CD55 and CD59 manifests as a complement-mediated hemolysis of PNH red blood cells (RBCs). In untreated patients, the absence of CD59 allows the formation of the MAC to proceed unchecked [1,3]. The MAC is the ultimate product of the terminal complement cascade, forming pores in the target cell’s membrane to cause direct lysis [4]. In PNH, this process predominantly affects RBCs, leading to their destruction within blood vessels, commonly known as ‘intravascular hemolysis’ (IVH) [1,3].

Untreated PNH patients may present multiple clinical symptoms, most commonly fatigue, anemia, dyspnea, hemoglobinuria, and smooth muscle dystonia [1,5,6,7]. Importantly, these patients have a high risk of serious complications; ~40% may experience thrombosis, the leading cause of death in untreated PNH patients [8,9,10], while further long-term complications can include pulmonary hypertension and kidney dysfunction [11,12,13].

The availability of complement C5 inhibitors for the treatment of PNH has altered the natural course of the disease. By blocking the cleavage of C5 and downstream MAC formation, C5 inhibitors are typically effective at controlling IVH [3]. As a result, the rates of thrombosis and other complications have improved compared to the pre-complement inhibitor era [5,11,13], leading to survival in PNH patients treated with C5 inhibitors approaching that of an age- and sex-matched general population [5].

**Figure 1:**
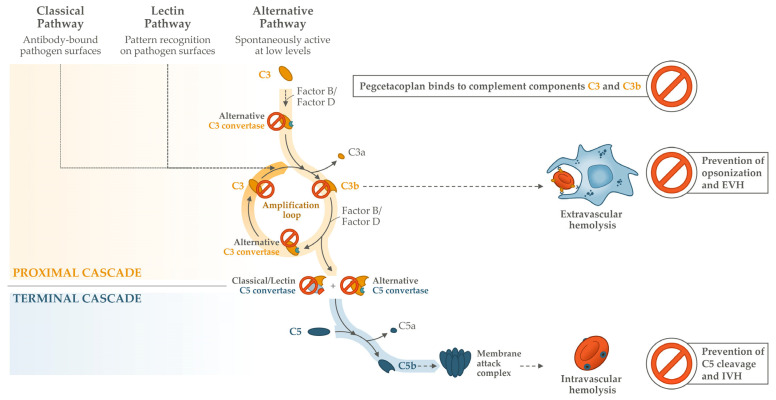
The complement system and mechanism of action of pegcetacoplan. Dashed arrows represent processes with several steps. EVH, extravascular hemolysis; IVH, intravascular hemolysis.

However, hemolysis in PNH can occur in two forms, because CD55 and CD59 act as checkpoints at different levels of the complement cascade. In the absence of CD55, an important negative regulator of C3 convertase is lost [1,3]. C3 convertases are pivotal enzymes in the proximal complement cascade, representing the point at which all complement cascade pathways converge to cleave C3 into its active components C3a and C3b [4]. In addition to C3b triggering downstream complement cascade activity, it also acts directly as an opsonin—a complement fragment that labels target cells for recognition, ingestion, and destruction by phagocytic cells [14]. Without CD55, C3b opsonins accumulate on the surface of PNH RBCs, leading to their phagocytosis by macrophages in the spleen or liver, commonly known as ‘extravascular hemolysis’ (EVH) [1,3] (Figure 1). While C5 inhibitors block terminal complement cascade activity to compensate for the loss of MAC-regulating CD59, they do not address the loss of CD55, which functions upstream of their C5 target [3].

The emergence and clinical relevance of EVH, which is typically inconspicuous in untreated PNH patients, may be viewed as an unintended mechanistic consequence of C5 inhibition [15,16]. Indeed, the mean proportion of PNH RBCs that are positive for C3 fragment deposition (an indicator of EVH) is markedly increased in PNH patients on C5 inhibitor therapy compared to untreated PNH patients [17,18]. Thus, although C5 inhibitors have improved the prognosis for PNH patients by reducing serious complications, a high proportion of patients exhibit clinical signs of EVH or residual IVH during stable C5 inhibitor therapy [19]. Up to 91% of patients have ongoing anemia [20,21], up to 89% still report fatigue [20,21], and up to 52% remain transfusion-dependent [21]. Furthermore, the reported frequency of many other PNH symptoms, such as dyspnea, cognitive problems, and pain, remain unchanged from the time of diagnosis to during C5 inhibitor therapy [20]. It was in this setting that inhibitors of the proximal complement cascade were viewed as a promising next generation of complement inhibitor therapy for PNH [3], both as a potentially more effective treatment for newly diagnosed patients and also as a novel option for patients with a suboptimal response to C5 inhibition.

This article provides a comprehensive overview of pegcetacoplan, the first approved proximal complement inhibitor for treating PNH, describing how its strong efficacy is linked to its mechanism of action. Furthermore, additional data and learnings obtained from over three years of experience with pegcetacoplan are summarized from disparate sources, including long-term efficacy and safety results, real-world clinical experiences, pharmacokinetic characteristics, and extensive practical guidance. This summary may support PNH-treating physicians in their clinical decision-making, particularly at a time when pegcetacoplan has recently become available to a larger PNH patient population across Europe.

## 2. Pegcetacoplan Addresses Both IVH and EVH, Providing Broad Hemolysis Control and Improving a Range of Hematological Markers of PNH

Pegcetacoplan was the first targeted inhibitor of C3 and its active cleavage product C3b available for patients with PNH. The US Food and Drug Administration (FDA) approved pegcetacoplan in 2021 for the treatment of adults with PNH [22], shortly followed by the European Medicines Agency (EMA)’s approval of it in 2021 for adults with PNH who had anemia after at least 3 months of C5 inhibitor therapy. A recent expansion to its indication by the EMA has made pegcetacoplan available for the treatment of both complement inhibitor-naïve and -treated adults with PNH who have hemolytic anemia [23].

By acting at a central point in the complement cascade, pegcetacoplan addresses both the proximal and terminal complement dysregulation characterizing PNH RBCs. Pegcetacoplan binds to C3 and C3b, directly inhibiting both C3 and C5 convertases to potentially provide more than one route of complement pathway blockade. The inhibition of C3 cleavage by a direct binding to C3 affects all pathways of complement activation, and the inhibition of C3 cleavage by binding to the C3b subunit of C3 convertase specifically targets the alternative complement pathway [24]. These mechanisms prevent the proximal cascade-mediated opsonization of CD55-deficient RBCs, blocking the pathway to EVH. In addition, pegcetacoplan binds the C3b subunit of C5 convertases from both classical/lectin and alternative pathways, blocking the downstream effects of the terminal complement cascade to simultaneously control the IVH caused by the loss of CD59 [24].

**Figure 2:**
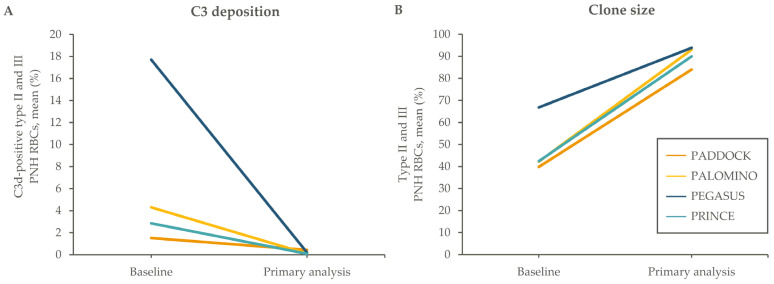
Change from baseline in (**A**) C3 deposition and (**B**) RBC clone size upon pegcetacoplan treatment. Patients in PADDOCK, PALOMINO, and PRINCE were complement inhibitor-naïve, while PEGASUS enrolled patients with a suboptimal response to complement C5 inhibitor treatment. Only patient-level data for the PHAROAH study were reported, so these are not included here. The primary analysis for PADDOCK and PALOMINO occurred at Day 365, for PRINCE it occurred at week 26, and for PEGASUS it occurred at week 16 of pegcetacoplan treatment. PNH, paroxysmal nocturnal hemoglobinuria; RBC, red blood cell.

This comprehensive mechanism of action has been directly demonstrated in PNH patients. In pegcetacoplan clinical trials, C3 deposition on PNH RBCs declined rapidly upon treatment to become almost completely abrogated, pointing to a robust blockade of C3b-mediated opsonization and subsequent EVH [25,26] (Figure 2A). Similarly, a measure of the effective control over IVH and EVH is the proportion of total RBCs in circulation that derive from a PNH mutant clone, often termed the ‘PNH RBC clone size’ [27]. PNH RBC clone sizes increase substantially upon pegcetacoplan therapy in complement inhibitor-naïve and C5 inhibitor-treated patients, indicating that PNH RBCs experience a broad level of protection from hemolysis [25,26,28] (Figure 2B).

The broad hemolysis control achieved with pegcetacoplan is confirmed by improvements across a wide range of hematological markers of PNH. Lactate dehydrogenase (LDH), classically elevated during IVH [29], was maintained at or reduced to normal or near-normal levels in patients previously treated with C5 inhibitors and in complement inhibitor-naïve patients who received pegcetacoplan, respectively [25,26,30]. A long-term follow-up showed that the median LDH was stable at normal levels for up to 3 years of pegcetacoplan treatment [31]. Beyond LDH, pegcetacoplan also durably improves hematological markers indicative of EVH, such as absolute reticulocyte count (ARC) and bilirubin [29]. Across pegcetacoplan clinical studies, the mean ARC was reduced to normal levels upon treatment with pegcetacoplan, indicating a reduced requirement for bone marrow to compensate for PNH-related hemolysis [25,26,30,31], while the mean bilirubin was similarly reduced to normal levels (defined as a total bilirubin < 1.1 mg/dL), indicating reduced RBC breakdown in the reticuloendothelial system [25,31,32] (Table 1). The mean ARC and indirect bilirubin were both maintained below the upper limit of normal (ULN) for up to 3 years of follow-up, highlighting the durability of EVH control with pegcetacoplan [31].

## 3. Pegcetacoplan’s Mechanism of Action Translates to Sustained Clinical Benefits and Pegcetacoplan Is Well Tolerated in PNH Clinical Trials

Crucially, a proximal complement inhibitor must be able to demonstrate that its mechanism of action in instituting broad hemolysis control leads to meaningful improvement in PNH patients, particularly in terms of the major clinical signs of EVH: anemia, transfusion dependence, and fatigue.

In the Phase 3 PRINCE trial of adults with PNH who were complement inhibitor-naïve, pegcetacoplan was superior to supportive care (including transfusions, anticoagulants, corticosteroids, and supplements [iron, folate, and vitamin B12]) for the co-primary endpoint of a proportion of patients achieving hemoglobin stabilization (defined as the avoidance of a > 1 g/dL decrease in hemoglobin from baseline to week 26: 86% vs. 0%; *p* < 0.0001). The other co-primary endpoint for PRINCE was a change from baseline in LDH levels at week 26, for which pegcetacoplan was also superior to supportive care (−1871 U/L vs. −400 U/L; *p* < 0.0001). Patients in the pegcetacoplan arm had improved mean hemoglobin levels from 9.4 g/dL at baseline to 12.8 g/dL at week 26, and the least squares mean change from baseline in terms of hemoglobin was significantly higher in the pegcetacoplan arm than the supportive care arm (+2.9 g/dL vs. +0.3 g/dL; *p* = 0.0019). In the 12 months prior to PRINCE enrollment, six patients (17%) in the pegcetacoplan arm and four patients (22%) in the supportive care arm were transfusion-free. Over the 26-week randomized controlled period, 32 patients (91%) in the pegcetacoplan arm were transfusion-free compared to 1 patient (6%) in the control arm (*p* < 0.0001). Fatigue was measured using the Functional Assessment of Chronic Illness Therapy (FACIT)-Fatigue scale, where an increase of 5 points indicates a clinically important change in fatigue for PNH patients [35]. At week 26, patients in the pegcetacoplan arm had a mean change from baseline in their FACIT-Fatigue score of +7.8 compared to +3.3 in the control arm [30].

In the Phase 3 PEGASUS trial of adults with PNH who had hemoglobin < 10.5 g/dL despite eculizumab therapy, a difficult-to-treat population, pegcetacoplan was superior to eculizumab with respect to the primary endpoint of a change in hemoglobin level from baseline to week 16 (adjusted mean difference of +3.8 g/dL; *p* < 0.001). Patients in the pegcetacoplan arm had improved mean hemoglobin levels, from 8.7 g/dL at baseline to 11.5 g/dL at week 16. In the 12 months prior to PEGASUS enrollment, 10 patients (24%) in the pegcetacoplan arm and 10 patients (26%) in the eculizumab arm were transfusion-free. Over the 16-week randomized controlled period, 35 patients (85%) in the pegcetacoplan arm were transfusion-free compared to 6 patients (15%) in the eculizumab arm (*p* < 0.001). At week 16, patients in the pegcetacoplan arm had a mean change from baseline in their FACIT-Fatigue score of +9.2 compared to −2.7 in the eculizumab arm [28]. In an analysis of the PEGASUS trial at week 48, improvements in disease markers were sustained for patients in the pegcetacoplan arm. Patients initially randomized to the eculizumab arm for the 16-week randomized controlled period switched to the pegcetacoplan treatment for the open-label period; by week 48, patients who switched displayed hematological and clinical outcomes similar to those who had received pegcetacoplan from the study’s initiation [26].

Pegcetacoplan’s ability to address the major unmet needs associated with EVH or residual IVH was maintained over a long-term follow-up. In an analysis that integrated the data from the PEGASUS and PRINCE trials with their long-term open-label extension studies (OLE), the improvement in mean hemoglobin demonstrated by the parent trials was maintained for 3 years in PEGASUS patients and 2.5 years in PRINCE patients (the maximum follow-up reported for both studies). Similarly, annualized transfusion avoidance rates were maintained between 71 and 86% and mean FACIT-Fatigue scores were maintained at or close to the general population norm of 43.6 [36] across both patient populations [31].

The integrated analysis of PEGASUS and PRINCE trials with their OLEs also demonstrated the long-term safety of pegcetacoplan, identifying no new safety signals compared to the primary analyses of the parent trials, which found pegcetacoplan to be well tolerated. The most common treatment-emergent adverse events included hemolysis, COVID-19, diarrhea, headache, and nasopharyngitis [28,30,31]. Of these adverse events, acute breakthrough hemolysis (BTH) has been the topic of most discussion in the PNH community, largely because of hypothesis that the efficacy of proximal complement inhibition leading to a higher PNH RBC clone size may increase the risk of a hemolysis episode being severe [34]. A report characterizing clinically significant BTH during PEGASUS, PRINCE, and their ongoing OLE studies, found that, overall, these events occurred infrequently during pegcetacoplan therapy (exposure-adjusted incidence: 0.19 events per year) and around half of events were mild or moderate in severity [37].

While clinically significant BTH is associated with increased LDH during the event [37], indicating IVH, it is important to note that BTH on pegcetacoplan does not appear to have been associated with thrombosis, one of the most serious clinical complications of PNH, in the clinical trial setting, given the low thrombosis rates reported in the complete populations. Thrombosis was well controlled over the long-term follow-up, with only three patients (2%) having thrombotic events in the context of multiple associated comorbidities, or after discontinuation [31]. Indeed, in 409 patient-years of pegcetacoplan exposure across seven clinical trials, only five thrombosis events have been reported (two of which occurred in the same patient) [38]. Another common concern for PNH-treating physicians is the risk of serious infections with encapsulated bacteria in patients receiving complement inhibitor therapy. However, no cases of encapsulated meningococcal infection have occurred in pegcetacoplan clinical trials to date [38].

Pegcetacoplan has also been evaluated in post hoc analyses of several subpopulations of PNH patients with the aim of assessing its efficacy and safety in specific clinical situations. For example, patients with a hemoglobin level ≥ 10 g/dL at baseline of the PEGASUS and PRINCE studies (n = 33) experienced rapid and sustained improvements in their hematological parameters and an enhanced quality of life with pegcetacoplan over up to 3 years of follow-up, suggesting that PNH patients with mild anemia derive a similar benefit to those with moderate-to-severe anemia [39]. Similarly, patients with a history of aplastic anemia at baseline in the PEGASUS (n = 20) and PRINCE (n = 10) studies were found to experience benefits from pegcetacoplan comparable to those in patients with no history of aplastic anemia [40,41], while in a subset of PEGASUS (n = 14) and PRINCE patients (n = 9) with impaired bone marrow function at baseline (defined as hemoglobin < 10 g/dL and absolute neutrophil count < 1.5 × 10^9^ cells/L), pegcetacoplan was associated with greater improvements in hematological and clinical markers than eculizumab or supportive care, respectively [42].

## 4. A Growing Body of Real-World Evidence Supports the Strong Efficacy and Safety of Pegcetacoplan Shown in PNH Clinical Trials

OPERA, an ongoing prospective observational study of adults with PNH in the US, has reported baseline and follow-up data for real-world patients enrolled over 12–18 months to date. Mean hemoglobin levels, transfusion avoidance rates, patient-reported fatigue, and work productivity assessments were observed to improve with pegcetacoplan, with low rates of healthcare resource utilization [43,44,45,46].

The Adelphi PNH Disease Specific Programme™ surveyed 14 physicians from across Europe and the USA to collect data on 61 patients with PNH who had received pegcetacoplan in real-world clinical practice for ≥ 1 month. Improvements in their mean hemoglobin levels, physician-perceived fatigue, and health-related quality of life were reported [47].

A collaborative report on real-world pegcetacoplan use in UK and French specialist PNH centers described clinical outcomes in 48 patients, where pegcetacoplan was effective at improving or stabilizing hematological parameters after 3 months of therapy. Pegcetacoplan was well tolerated, but a small number of patients had repeated BTH events. However, the BTH was manageable, with most patients remaining on pegcetacoplan [48].

A group of Spanish investigators recently described the clinical and hematological results of pegcetacoplan treatment (median [range] 10 months [1–58]) in a series of 23 patients who had suboptimal responses to previous therapies. Comparing pre-pegcetacoplan to post-pegcetacoplan, improvements in hemoglobin levels, hemolysis parameters, transfusion avoidance, and quality of life were reported. Similarly to the UK and French study, a subset of patients experienced BTH, but all of these events were manageable with patients remaining on pegcetacoplan [49].

Although some of the real-world studies to date have included relatively small sample sizes, it is encouraging that the effectiveness of pegcetacoplan appears to be highly consistent between reports. Other case series and case reports describe similar outcomes after real-world pegcetacoplan therapy, including cases of pegcetacoplan use during pregnancy and its use peri-operatively (Table 2).

Finally, a large body of evidence from the worldwide post-marketing setting of pegcetacoplan has shown that thrombosis rates are low; over a total of 626 patient-years of pegcetacoplan exposure, the thrombosis rate was 0.32 events per 100 patient-years [50]. This is favorable compared to the thrombosis rates observed with C5 inhibitor therapy in PNH in the real world. In 509 UK patients treated with C5 inhibitors for a total exposure of 3130 patient-years, the thrombosis rate was 0.73 events per 100 patient-years [5]. The rates of meningococcal infection are similarly favorable. Over a total of 1127 patient-years of combined clinical and post-marketing pegcetacoplan exposure worldwide, there have been 0 events of encapsulated meningococcal infection reported up to November 2023 [50]. In the 509 UK patients treated with C5 inhibitors, the meningococcal infection rate was 0.35 events per 100 patient-years [5].

**Table 2:** Real-world evidence surrounding pegcetacoplan. ARC, absolute reticulocyte count; BTH, breakthrough hemolysis; CFB, change from baseline; EVH, extravascular hemolysis; FACIT, Functional Assessment of Chronic Illness Therapy; LDH, lactate dehydrogenase; PNH, paroxysmal nocturnal hemoglobinuria; PROMIS, Patient-Reported Outcomes Measurement Information System; QoL, quality of life; ULN, upper limit of normal.

	Study Type	Sample Size	Key Findings
**Adelphi PNH ** **Disease Specific ** **Programme™ ** **Europe (France, ** **Italy, Spain and ** **Germany) and USA [47]**	Non- interventional real-world survey with retrospective data collection	14 physicians and 61 patients	Improvement in hemoglobin levels: +2.5 g/dL mean change in hemoglobin from baseline to ≥ 1 month (n = 61, from 9.0 g/dL to 11.5 g/dL); +3.3 g/dL mean change in hemoglobin from baseline to ≥ 6 months (n = 23, from 8.3 g/dL to 11.6 g/dL). Physicians perceived fatigue to be less severe in their patients following pegcetacoplan treatment compared to baseline and also perceived improvements in health-related quality of life The majority of patients who switched from a C5 inhibitor were more satisfied with pegcetacoplan, which was mainly (76.9%) attributed to them experiencing less fatigue
**OPERA** **USA [43,44,45,46]**	Prospective, observational, opt-in study	54 patients	Improvement in hemoglobin levels, transfusion avoidance, and fatigue: +3.3 g/dL mean change in hemoglobin from baseline to latest follow-up (n = 39; from 8.8 g/dL to 12.1 g/dL; median [IQR] 7.2 months [6.1] since pegcetacoplan initiation) [43]. Change in transfusion avoidance rates from 41% (n = 16/39) in the 12 months prior to enrollment to 82% (n = 40/49) at last follow-up on pegcetacoplan (median [IQR] 9 months [4] of pegcetacoplan treatment) [45]. Mean change in FACIT-Fatigue score from 29.4 at baseline to 37.9 at 3 months (n = 8) [46]. Patients experienced low incidence rates of emergency room visits and hospitalizations Improvements in cognitive function (PROMIS T-score), work productivity and reduced activity impairment after initiating pegcetacoplan treatment
**Real-world ** **evidence** **UK/France [48]**	Retrospective service review	48 patients	Pegcetacoplan was effective at improving or stabilizing hematological parameters at 3 months in real-world patients experiencing anemia due to EVH after C5 inhibition: +2.2 g/dL mean change in hemoglobin (n = 41); −0.2 × ULN mean change in LDH (n = 37); −133 × 10^9^ cells/L mean change in ARC (n = 28). Six patients (13%) experienced BTH events outside of a clinical trial setting, with most events being associated with a decrease in hemoglobin Most patients remained on pegcetacoplan treatment during BTH management and events resolved within a mean of 16 days
**Real-world ** **experience** **Spain [49]**	Case series	23 patients	Improvement in hemoglobin levels, hemolysis parameters, transfusion avoidance, and patient-reported QoL: +3 g/dL change in median hemoglobin; −0.2 × ULN change in median LDH; −134 × 10^9^ cells/L change in median ARC; Change in transfusion avoidance rates from 35% (n = 8/23) in the 6 months prior to pegcetacoplan to 83% (n = 19/23) in the 6 months after pegcetacoplan initiation; Patients rated their improvement in quality of life highly (median [range] 4 [0–5] on a 5-point scale). Quick efficacy upon treatment initiation and an excellent safety profile BTH was reported in 7 patients (30%) in the 6 months preceding pegcetacoplan initiation compared to 3 patients (13%) during the first 6 months of pegcetacoplan treatment, and all of these events were manageable with patients remaining on pegcetacoplan
**Real-world ** **experience** **Spain [51]**	Case series	4 patients	Pegcetacoplan improved outcomes in patients with suboptimal response to C5 inhibitor treatment The effect of improvements in hemoglobin levels and control of underlying hemolysis led to transfusion independence and improvements in patient-reported outcomes
**Individual patient cases** **Italy, USA, Australia, and Spain [52,53,54,55,56,57,58]**	Case reports	1–2 patients per report	Several unique patient cases describing pegcetacoplan treatment for PNH in various circumstances, including in the settings of myeloproliferative neoplasm [52], aplastic anemia [53], a suboptimal response to C5 inhibition [54,55], patients receiving major surgeries [56], Budd–Chiari syndrome [57], and pregnancy [58]

## 5. Extensive Practical Guidance Supports the Real-World Clinical Use of Pegcetacoplan in PNH

**Figure 3:**
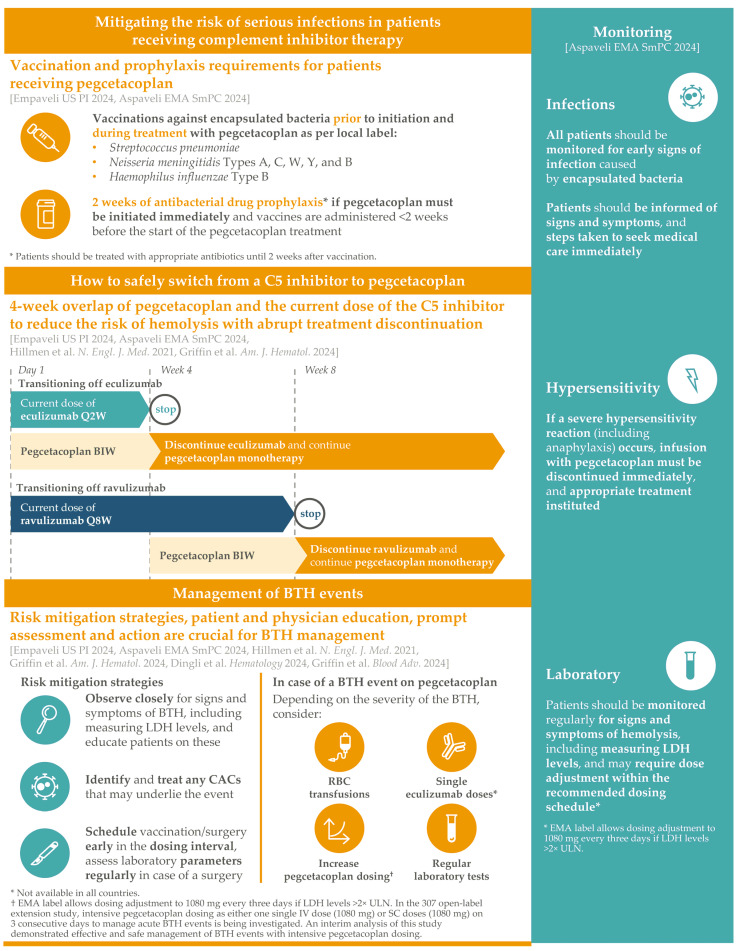
Practical guidance supporting the use of pegcetacoplan, derived from its prescribing information and peer-reviewed articles [22,23,28,48,63,64]. BIW, twice-weekly; BTH, breakthrough hemolysis; CAC, complement-amplifying condition; EMA, European Medicines Agency; IV, intravenous; LDH, lactate dehydrogenase; PI, Prescribing Information; Q2W; every 2 weeks; Q8W, every 8 weeks; RBC, red blood cell; SC, subcutaneous; SmPC, Summary of Product Characteristics; t1/2, half-life; ULN, upper limit of normal.

The optimal therapy in PNH requires a delicate balance; suppressing complement activity sufficiently to prevent chronic hemolysis and its serious complications while managing the potential risks associated with the therapeutic inhibition of a system that plays an important role in immune defenses [50]. Thus, it is critical that treating physicians are given sufficient guidance on how to manage the therapy’s initiation and monitoring and specific situations, while patient education on risks and warnings signs is imperative (Figure 3).

Common to all complement inhibitors is the risk of serious infections with encapsulated bacteria; to mitigate this, patients should receive appropriate vaccinations prior to initiating complement therapy. Guidance on implementing these protective measures is available as part of pegcetacoplan’s prescribing information [22,23]. Patients must be vaccinated against *Neisseria meningitidis*, *Streptococcus pneumoniae*, and *Haemophilus influenzae* according to local guidelines at least 2 weeks prior to initiating pegcetacoplan, or if immediate therapy is indicated, vaccinations should be administered as soon as possible and the patient treated with antibiotics until 2 weeks after vaccination. Patient education on monitoring for early signs of infection is also encouraged [22,23]. So far, no meningococcal infections with encapsulated bacteria have been reported with pegcetacoplan to date, suggesting effective risk mitigation [50].

Once a patient’s vaccination status is confirmed, pegcetacoplan’s product information also provides clear instructions on how to switch to pegcetacoplan therapy for those patients currently receiving a C5 inhibitor [22,23]. A 4-week overlap period is recommended because pegcetacoplan can achieve steady-state serum concentrations in this timeframe, allowing physicians to discontinue C5 inhibition safely. Once on stable monotherapy, pegcetacoplan is administered subcutaneously twice a week at 1080 mg, with self-administration and home infusion possible for patients who have received training and tolerated the treatment well under supervision [22,23]. In a real-world post-marketing setting, 96% of patients reported feeling confident in self-administering pegcetacoplan after receiving training, with an estimated compliance rate of 97% [50], confirming the high adherence observed over 48 weeks of pegcetacoplan’s open-label extension study (98%) [59].

**Figure 4:**
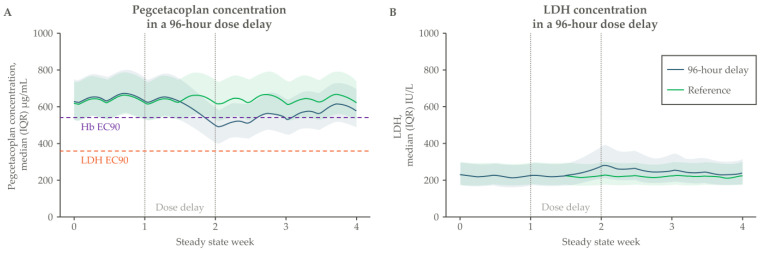
Population pharmacokinetic modeling of (**A**) pegcetacoplan concentration and (**B**) LDH level with a 96 h dose delay. The dose delay analysis was performed using a population pharmacokinetic model [60] (see Appendix A) and assuming a steady-state concentration of pegcetacoplan at a SC dose of 1080 mg twice weekly. EC, effective concentration; Hb, hemoglobin; IQR, interquartile range; LDH, lactate dehydrogenase; SC, subcutaneous.

However, for any therapy, the possibility cannot be excluded that patients might occasionally forget to take a dose on time, while activities such as travel may interrupt usual administration routines. Pegcetacoplan’s predicted median effective half-life of elimination of 8.6 days, assessed by population pharmacokinetic analysis [60], contributed to the low variability in the steady-state serum concentrations of pegcetacoplan during the randomized controlled period of the PEGASUS trial [28], a population with difficult-to-control PNH. Population pharmacokinetic modeling also showed minimal peak-to-trough variation in pegcetacoplan concentrations at a steady state, leading to a consistent exposure predicted to exceed the EC90 for hemoglobin and LDH response in approximately 75% of PNH patients. Factors such as age, sex, ethnicity, and renal and hepatic function had no meaningful impact on pegcetacoplan exposure, and predicted steady-state exposure exceeds the thresholds required for robust hemoglobin and LDH responses in patients with a high body weight [60,61]. Furthermore, modeling has predicted that at steady state, a single dose delayed for up to 96 h would not meaningfully affect the pegcetacoplan serum concentration or LDH levels (Figure 4), suggesting that occasional unavoidable deviation from the recommended administration routine is unlikely to have safety implications (see Appendix A for methods of pharmacokinetic and pharmacodynamic modeling).

Another aspect common to all patients treated with any complement inhibitor therapy is the risk of acute BTH [48]. Two mechanisms for BTH were established based on observations of patients treated with C5 inhibitors; pharmacokinetic BTH, where serum concentrations of the complement inhibitor are inadequate to block terminal cascade activity, and pharmacodynamic BTH, where complement-amplifying conditions (CACs) triggered by infection or inflammation lead to excess terminal cascade activity despite adequate serum drug concentrations [3]. In a recent description of hemolysis adverse events occurring during the PEGASUS study, 62% of events had a potential CAC identifiable from adverse event reporting and/or medical records [62]. These data suggest that the majority of hemolysis events had a possible pharmacodynamic influence on this challenging patient population, for whom the PEGASUS study represented one of the first opportunities to manage suboptimal responders to C5 inhibition with a novel proximal complement inhibitor therapy in PNH. Most hemolysis events in PEGASUS were manageable without pegcetacoplan discontinuation (65%) [62]. In PRINCE, which studied a population who were naïve to prior complement inhibitor therapy, no acute hemolytic events were reported over 26 weeks of pegcetacoplan treatment [30].

Currently, pegcetacoplan is the only proximal complement inhibitor with strategies in place for managing BTH events [48], although generalized guidance on managing pharmacodynamic BTH is available [63]. Clinical trials have demonstrated the effective management of clinically significant BTH events during pegcetacoplan; in addition to pegcetacoplan dose escalations [59,62], transfusions were utilized in 53% of events (n = 33/62) and C5 inhibitor therapy utilized in 10% (n = 6/62) [37]. Expert real-world experience with pegcetacoplan in the UK and France also found that BTH was manageable through dose modification, with the majority of patients being able to remain on pegcetacoplan. These expert groups jointly published an algorithm to advise on the diagnosis, intervention, and follow-up actions required for BTH while on pegcetacoplan [48]. Pegcetacoplan’s approved indication for PNH allows for a dose adjustment to 1080 mg every third day if a patient has LDH > 2 × ULN [23]. In addition, further management strategies are under investigation in the form of intensive subcutaneous or intravenous pegcetacoplan dosing. An interim analysis found that subcutaneous dosing on 3 consecutive days or a single intravenous dose led to the rapid improvement of LDH and hemoglobin in patients with acute BTH, with a median time to BTH resolution of 15 days [64], potentially offering even more options to physicians for safely and effectively navigating BTH events. 

## 6. Pegcetacoplan Enables PNH Patients to Aim for Better Outcomes: Conclusions

In terms of proximal complement inhibitors, new treatment options have emerged that aim to maintain the benefits of C5 inhibitors while also improving additional clinical and hematological features. Pegcetacoplan, the first approved proximal complement inhibitor for treating PNH, demonstrated its ability to improve the key hematological markers of both IVH and EVH compared to eculizumab in the Phase 3 PEGASUS study and compared to supportive care in the Phase 3 PRINCE study [26,28,30]. Furthermore, pegcetacoplan has substantial long-term clinical trial data supporting its sustained efficacy in PNH patients [31], as well as a growing body of real-world experiences complementing these positive trial results. Pegcetacoplan has also been shown to be well tolerated over long-term follow-ups [31] and, crucially, is not associated with risks of thrombosis and infection greater than those observed for C5 inhibitors when proper precautions are taken [50].

Thus, the expectations for what constitutes a response to therapy in PNH can now be raised. Pegcetacoplan has surpassed the previous limits that existed to improving patient quality of life [19,65], most notably demonstrated by pegcetacoplan’s impact on reducing fatigue. With the availability of more effective new treatment options comes the requirement of more fully embracing PNH as a heterogeneous, pleiotropic disease that requires individual patient assessments to understand ongoing symptoms and quality of life impairments [66]. Evaluating PNH patients in this way will enable a more personalized approach to therapy, rather than an approach focused on addressing terminal complement dysregulation and IVH alone.

Future large-scale real-world evidence generation efforts will provide important further insights on pegcetacoplan. The COMPLETE study is an ongoing, global, multicenter observational Phase 4 study that will describe the real-world effectiveness and safety of pegcetacoplan in the treatment of adult patients with PNH. COMPLETE will prospectively collect hematological clinical data, as well as healthcare resource use information and patient perspectives on quality of life and treatment satisfaction [67]. The International PNH Interest Group (IPIG) Registry will collect data from PNH patients receiving any therapy, including pegcetacoplan, as well as from patients not on complement inhibition [68].

In summary, the 3 years since its initial approval have confirmed pegcetacoplan as a valuable treatment option for adults with PNH, with long-term clinical trial data and real-world evidence to support its strong efficacy and safety in a broad patient population.

## Figures and Tables

**Table 1:** Hematological markers in pegcetacoplan-treated patients in the PADDOCK, PALOMINO, PEGASUS, and PRINCE studies. Patients in PADDOCK, PALOMINO, and PRINCE were complement inhibitor-naïve, while PEGASUS enrolled patients with a suboptimal response to complement C5 inhibitor treatment. Data from the PHAROAH study were excluded due to a lack of population-level data. ARC, absolute reticulocyte count; LDH, lactate dehydrogenase; NR, not reported; SD, standard deviation.

	PADDOCK [25,33]		PALOMINO [25,33]		PRINCE [30,32]		PEGASUS [26,32,34]		
	Baseline	Primary Analysis (Day 365)	Baseline	Primary Analysis (Day 365)	Baseline	Primary Analysis (Week 26)	Baseline	Primary Analysis (Week 16)	Follow-up Analysis (Week 48) *
**n at baseline ^†^**	22		4		35		41		
**Hemoglobin** Mean (SD), g/dL	8.5 (1.8)	12.1 (2.0)	7.7 (0.9)	13.0 (2.2)	9.4 (1.4)	12.8 (2.1)	8.7 (1.1)	11.5 (2.0)	11.3 (1.8)
**LDH** Mean (SD), U/L	2354.9 (988.0)	306.5 (324.7)	2548.8 (631.1)	226.0 (27.0)	2151.0 (909.4)	204.6 (90.0)	257.5 (97.6)	189.1 (78.1)	222.7 (141.1)
**ARC** Mean (SD), × 10^9^ cells/L	198.2 (63.0)	96.4 (33.4)	238.3 (91.0)	94.0 (26.9)	230.2 (81.0)	101.2 (30.8)	217.5 (75.0)	77.1 (26.6)	80.0 (26.8)
**Indirect bilirubin** Mean (SD), mg/dL	NR	NR	NR	NR	2.2 (1.1)	0.7 (0.5)	2.1 (1.8)	0.8 (0.9)	NR

* Only includes patients who were initially randomized to the pegcetacoplan arm (not patients who switched from eculizumab to pegcetacoplan for the open-label period of the study). ^†^ Patient numbers at primary or follow-up analysis may have been different due to data availability.

## Data Availability

Data are not in a publicly available repository. Sobi is committed to the responsible and ethical sharing of data at the participant level and summary data for medicines and indications approved by the EMA and/or FDA, while protecting individual participant integrity and compliance with applicable legislation. Data access will be granted in response to qualified research requests. All requests are evaluated by a cross-functional panel of experts within Sobi, and a decision on sharing will be based on the scientific merit and feasibility of the research proposal, the maintenance of personal integrity, and a commitment to the publication of the results. To request access to the study data, a data sharing request form (available at www.sobi.com (accessed on 6 August 2024)) should be sent to medical.info@sobi.com. Further information on Sobi’s data sharing policy and process for requesting access can be found at: https://www.sobi.com/en/policies (accessed on 6 August 2024).

## Appendix A

### Population Pharmacokinetic, Pharmacodynamic, and Dose Delay Modeling

The population pharmacokinetic analysis of pegcetacoplan included pooled data from 11 clinical studies, including the Phase 3 PEGASUS and PRINCE studies. The pooled studies included a range of subjects: healthy adults (n = 108), adults with severe renal impairment and matched controls with normal renal function (n = 8 of each), or adults with paroxysmal nocturnal hemoglobinuria (n = 160, including eculizumab-naïve or those remaining anemic despite ≥ 3 months of eculizumab treatment). A range of dosing regimens were also included: pegcetacoplan was administered via a subcutaneous bolus injection, subcutaneous delivery devices, or intravenous infusion, and doses ranged from a single 25 mg subcutaneous injection to once-weekly 2600 mg subcutaneous infusions [60].

The population pharmacokinetic model was used to predict pegcetacoplan and LDH concentrations over time under conditions of a dose delay at steady state and to quantify the impact of intrinsic and extrinsic factors on pegcetacoplan exposure.

The pharmacodynamic analysis of pegcetacoplan included pooled data from five clinical studies of adults with paroxysmal nocturnal hemoglobinuria, including the Phase 3 PEGASUS and PRINCE studies. Simulations were performed (including intrinsic and extrinsic factors as prespecified covariates) to evaluate the predicted hemoglobin and lactate dehydrogenase responses to subcutaneous injections of pegcetacoplan at 1080 mg twice weekly and sigmoidal Emax models were employed to describe hemoglobin and lactate dehydrogenase concentrations as a function of a median steady-state pegcetacoplan serum concentration [60,61].
